# Revocable Identity-Based Matchmaking Encryption with Equality Test for Smart Healthcare

**DOI:** 10.3390/s25154588

**Published:** 2025-07-24

**Authors:** Xiaokun Zheng, Dong Zheng, Yinghui Zhang

**Affiliations:** 1 College of Computer, Qinghai Normal University, Xining 810008, China; xiaokzheng@163.com; 2 National Engineering Research Center for Secured Wireless, Xi’an University of Posts and Telecommunications, Xi’an 710121, China; zhengdong@xupt.edu.cn

**Keywords:** revocable, identity-based matchmaking encryption, equality test, smart healthcare

## Abstract

Smart healthcare establishes a safe, reliable, and efficient medical information system for the public with the help of the Internet of Things, cloud storage, and other Internet technologies. To enable secure data sharing and case-matching functions in smart healthcare, we construct a revocable identity-based matchmaking encryption with an equality test (RIBME-ET) scheme for smart healthcare. Our scheme not only ensures the confidentiality and authenticity of messages and protects the privacy of users, but also enables a cloud server to perform equality tests on encrypted ciphertexts from different identities to determine whether they contain the same plaintext and protects the confidentiality of data in the system through a user revocation mechanism. Compared with the existing identity-based encryption with equality test (IBEET) and identity-based matchmaking encryption with equality test (IBME-ET) schemes, we have improved the efficiency of the scheme and reduced communication overhead. In addition, the scheme’s security is proven in the random oracle model under the computational bilinear Diffie–Hellman (CBDH) assumption. Finally, the feasibility and effectiveness of the proposed scheme are verified by performance analysis.

## 1. Introduction

Smart healthcare [1,2,3] is a new medical model that uses cloud storage, cloud computing, big data, and other information technologies to improve people’s health. It provides complete data support for patients and doctors and enables telemedicine, intelligent monitoring, data analysis, and other medical services. The purpose of smart healthcare is to use the user’s medical data to complete relevant medical operations and to provide more intelligent services.

Smart healthcare can effectively achieve optimal allocation of resources and can improve the medical level of local hospitals, but under the smart healthcare system, sensitive medical data transmission is involved. This poses a challenge to how smart healthcare can be used to store and manage data more effectively. Smart healthcare systems face several security issues, such as data origin and integrity verification; data confidentiality and user privacy protection; and key leakage issues. The complexity of security issues facing smart healthcare makes it crucial to design a secure and efficient data encryption scheme. Especially in systems where there may be users who do not want sensitive medical data to be leaked to other unrelated personnel, there is a need to achieve user privacy protection while securing medical data.

The most common method for securing data is encryption, which ensures the confidentiality of the data. We aim to achieve secure sharing of medical data and matching of patient cases to help patients communicate better. Lin et al. provided a diversified butterfly attractors of memristive Hopfield neural network (HNN) with two memristive systems, which can successfully realize the privacy protection of medical data [4]. Ding et al. proposed a novel chaotic memristive neural network (MNN) that integrates two memristors into a traditional Hopfield neural network [5]; the secure algorithm was then successfully applied in a remote sensing system to protect image data privacy. Although chaotic encryption protects data privacy, public key encryption is more suitable for implementing fine-grained access control, data sharing, and case matching of users in smart healthcare. Yang et al. [6] proposed public key encryption with equality test (PKEET) for use in Internet-based systems for private health records (PHRs) [7,8,9], which enables the cloud server to perform an equality test of two encrypted ciphertexts to confirm whether they contain the same plaintext, and the cloud server helps the patient to match the their data with that of others. When uploading data in a smart healthcare system, it is important to ensure the authenticity of the user’s data during data uploading to prevent other malicious users from posting malicious or false medical information.

In recent years, several scholars have shifted their research focus to identity-based encryption with equality test schemes (IBEET). IBEET eliminates certificate management issues in the PKEET, and IBEET is used in several applications, such as in smart healthcare and Internet of Vehicles (IoV) road monitoring. However, there are some security issues with IBEET, since the current IBEET [10,11] does not consider the anonymity of both the sender and the receiver, which may lead to the disclosure of identity information. The identity-based matchmaking encryption (IBME) scheme investigated by Ateniese et al. [12] provides an enhanced privacy protection mechanism for user matching in smart healthcare. IBME provides a bilateral access control function, which allows the sender and receiver to specify each other’s respective identities at the same time. The IBME scheme prevents the leakage of identity privacy, and when there is a mismatch, except for decryption failures, the receiver cannot access arbitrary information. In addition, user revocation [13,14,15] is also an issue that needs to be considered in intelligent medical systems; when the user’s key is leaked or lost, an attacker can obtain the user’s key and decrypt the ciphertext sent by the sender to obtain the corresponding medical information, leading to the leakage of the user’s private information.

In smart healthcare, the user can issue a query request to the server to help contact other users with the same disease, for example, two patients with different doctors but the same symptoms wanting to seek each other’s experience and help. One question here is how bilateral access control can be realized [16] to better protect the privacy of both parties. The doctor and patient designate each other; the doctor encrypts the data for a patient, and only the patient can successfully decrypt it. When the two parties do not match, there is no information leakage except for decryption failure. However, the traditional IBEET scheme does not solve this problem.

To address the challenges of protecting sender and receiver privacy, enabling bilateral access control, preserving data authenticity, and mitigating key loss and disclosure risks, we propose a revocable identity-based matchmaking encryption with equality test (RIBME-ET) scheme. This solution integrates identity-based matchmaking encryption (IBME) with an equality test mechanism. Our scheme achieves user revocation and uses less time in the decryption, trapdoor generation, and equality test phases compared to the existing identity-based matchmaking encryption with equality test scheme (IBME-ET) [17]. Compared to the existing IBEET [10,18] scheme, our scheme enables user revocation, achieves finer-grained access control, and further enhances user privacy. The main contributions of this paper are as follows:

- We establish the first formal definition and security model for RIBME-ET, along with its concrete construction. The scheme implements a privacy-enhanced bilateral access control mechanism tailored to user matching in smart healthcare systems, ensuring both medical data authenticity and user privacy preservation.
- Cloud servers leverage the equality test functionality to compare ciphertexts in smart healthcare scenarios, enabling patients to seek mutual assistance and share experiences securely. Our design incorporates user revocation, safeguarding encrypted medical data from decryption even if keys are compromised or lost. Comprehensive security proofs and performance evaluations validate the scheme’s robustness and efficiency.

Organization of the paper: Here, we outline the structure of the rest of the paper. Section 2 introduces some related works. Preliminaries and definitions are described in Section 3 and Section 4, and we propose a security model for our scheme. In Section 5, we construct a RIBME-ET scheme. In Section 6, we prove the security of our scheme. Performance evaluations are presented in Section 7. Finally, we conclude this paper in Section 8.

## 2. Related Work

In smart healthcare, to ensure data confidentiality, patients encrypt their medical data and store it in an encrypted form on cloud servers. When one of these patients, Alice, wants to find other patients with the same condition as her to share their experiences, Alice will commission a third party to help her find such patients. At the same time, considering privacy, Alice will not directly disclose her disease information to the third party but will encrypt her disease information and send it to the third party. When another patient, Bob, also wants the third party to help him find other patients with the same condition, Bob similarly encrypts his medical information and sends it to the third party. Subsequently, the third party performs equivalence testing on the encrypted disease information without decrypting it. If Alice’s encrypted message and Bob’s encrypted message are both encrypted from the same disease information, the third party will notify Alice and Bob. It is important to note that the third party does not obtain this disease information. Traditional identity-based encryption and searchable encryption cannot meet the specific requirements of this application scenario. Encryption schemes encrypt information, and without the decryption key, it is impossible to perform computational processing on the ciphertext and therefore impossible to distinguish the relationships between these ciphertexts. In searchable encryption schemes, the ciphertext used for retrieval and the trapdoor sent by the user must be generated using the same public–private key pair. If the ciphertext and trapdoor used for retrieval are encrypted using different public–private key pairs, the ciphertext cannot be identified.

Yang et al. proposed a public key encryption scheme supporting equality testing (PKEET), which allows anyone to determine whether two ciphertexts generated under different public keys contain the same message [6]. This scheme provided a solution for case matching and user classification in smart healthcare. Tang et al. proposed a fine-grained authorization PKEET scheme that allows an authorized equality test to be conducted on ciphertexts by two users [19]. The scheme effectively improves PKEET authorization. Then Tang proposed the all-or-nothing PKEET scheme (AON-PKEET) [20], which specifies who can perform an equality test on ciphertexts. Moreover, Tang proposed the PKEET scheme with authorization of different granularity (ADG-PKEET) [21]. This scheme is an extension of FG-PKEET, which protects against offline message recovery attacks (OMRAs) through the dual-server mechanism.

Ma et al. proposed an equality test scheme with flexible authorization (PKEET-FA). This cryptographic scheme provides authorization based on four different scenarios [22], and the security of these cryptosystems is based on the difficult assumption of bilinear pairs. Huang et al. proposed public key encryption with an authorized equality test (PKE-AET), which employs cipher-level authorization and user-level authorization to enhance privacy protection [23], and this scheme allows for a comparison of specific users’ ciphertexts or all ciphertexts. Hassan et al. proposed an efficient certificateless public key encryption scheme with authorized equality tests in healthcare environments [24]. Susilo et al. proposed public key encryption with a multi-ciphertext equality test in cloud computing [25]. Ma et al. proposed efficient public key encryption with an outsourced equality test for cloud-based IoT environments [26].

An increase in users in the cloud environment may lead to an increase in certificate management burden. To better enable PKEET to be better applied to the cloud environment, Ma et al. combined identity-based encryption and an equality test [18] and proposed, for the first time, an identity-based encryption scheme with an equality test function (IBEET). Lee et al. proposed a semi-generic construction of public key encryption and identity-based encryption with equality test [10]. Xiong et al. constructed the notion of identity-based signcryption with equality test (IBSC-ET) by combining signcryption and an equality test scheme [27]. Yang et al. proposed an efficient identity-based encryption with an equality test in cloud computing [28].

Ateniese et al. proposed a new encryption scheme termed identity-based matchmaking encryption (IBME) [12]. This scheme enables both senders and receivers to specify the identity conditions that the other party must satisfy to decrypt the message. The main security guarantee is privacy-preserving identity matching: during the decryption process, when the sender and receiver do not match each other, no information will be revealed. IBME opens up new ways of secretly communicating and enables several new applications. IBME ensures message confidentiality and authenticity in a non-interactive manner and provides the functionality required for bilateral access control of identities. Therefore, IBME offers users a more convenient and secure communication approach.

Chen et al. proposed an IBME from standard assumptions in the standard model [29]. Jiang et al. proposed a revocable IBME in the standard model, whose security is reduced to the hardness of the decisional bilinear Diffie–Hellman problem and computational Diffie–Hellman problem [30]. Wu et al. proposed a fuzzy IBME and implemented it [31]. Yan et al. proposed an IBME-ET and proved its security under the random oracle [17].

As shown in Table 1, except for the IBME scheme, all other schemes achieved CCA security. The traditional PKEET [6] scheme implements a ciphertext equality test but does not consider user revocation and finer-grained access control features. The IBEET [10] scheme accomplishes identity substitution via public keys as opposed to the PKEET scheme but does not consider user revocation and bilateral access control. Recent IBME [12] schemes have achieved finer-grained access control from the sender to the receiver but have not implemented the ciphertext equality test. In contrast, the IBME-ET [17] scheme implements the ciphertext equality test and bilateral access control and does not consider key revocation. Compared with the above schemes, our scheme not only improves the efficiency of decryption and trap generation but also considers user revocation, so it is better able to protect data in smart healthcare.

## 3. Preliminaries

In this section, we review preliminaries including notations, bilinear maps, and the computational bilinear Diffie–Hellman (CBDH) assumption.

### 3.1. Bilinear Map

Given two cyclic groups $G_{1}$ and $G_{2}$ of prime order *p*, let *g* be a generator of $G_{1}$ and an admissible bilinear mapping *e*: $G_{1}$×$G_{1}$→$G_{2}$ is expected to satisfy the following properties:

- Bilinearity: $e\left({g_{1}}^{x},{g_{2}}^{y}\right)=e{\left(g_{1},g_{2}\right)}^{xy}$ for ∀$g_{1},g_{2}\in G_{1}$, and $x,y\in Z_{p}^{*}$.
- Non-degeneracy: e(g,g)≠1.

We say that $G_{1}$ is a bilinear group if the group operation in $G_{1}$ and the bilinear map *e*: $G_{1}$×$G_{1}$→$G_{2}$ are both efficiently computable.

### 3.2. Computational Bilinear Diffie–Hellman Assumption

Given a random tuple $\left(g,g^{a},g^{b},g^{c}\right)$ of a CBDH problem, where *g* is the generator of group $G_{1}$ and a,b, and *c* are randomly chosen from $Z_{p}^{*}$, its solution is $e{\left(g,g\right)}^{abc}$. Formally, the CBDH problem is hard in $\left(G_{1},G_{2},e\right)$ if, for every PPT adversary *A*,

$Pr\left[A\left(q,G_{1},G_{2},e,g,g^{a},g^{b},g^{c}\right)=e{\left(g,g\right)}^{abc}\right]\leq negl\left(\lambda \right)$.

We say that the CBDH assumption holds in $\left(g,G_{1},G_{2},e\right)$ if no PPT algorithm can compute $e{\left(g,g\right)}^{abc}$ with a non-negligible advantage.

## 4. Definition

In this section, we formalize the syntax and security of the RIBME-ET scheme.

### 4.1. System Model

**Figure 1:**
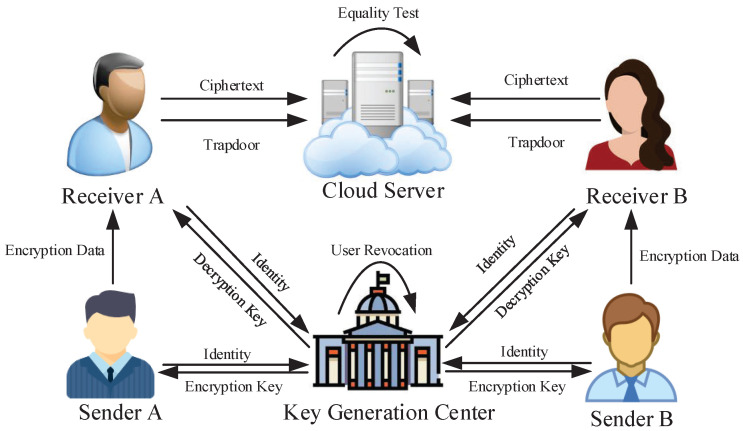
System model.

The system model is shown in Figure 1. The system model consists of the following four entities, namely the sender, receiver, key generation center (KGC), and cloud server, as listed below:

- Sender: The sender encrypts the messages with the encryption key and the specified receiver’s identity and then sends the resulting ciphertexts to the receiver.
- Receiver: The receiver can decrypt the ciphertexts, generate trapdoors, and upload both the ciphertexts and computed trapdoors to the cloud server.
- KGC: KGC is responsible for managing users in the system and generating encryption and decryption keys for senders and receivers. For user revocation, the KGC manages the distribution of the key; if the KGC stops sending the decryption key to the receiver, it means that the user has been revoked. Decryption algorithms and trapdoor algorithms require a decryption key that is related to the time period *t*.
- Cloud server: This entity is responsible for storing ciphertexts and providing equality tests.

### 4.2. Syntax of RIBME-ET

RIBME-ET is composed of the following polynomial algorithm:

- Setup$\left(1^{\lambda }\right)\to \left(mpk,msk\right)$: Input the security parameter λ, and the algorithm outputs a time period *t* as input to produce the system’s master public key mpk and master secret key msk. Then the mpk is used as an implicit input for the following algorithms.
- SKGen$\left(msk,ID_{Snd}\right)\to ek_{ID_{Snd}}$: After inputting the master secret key msk and the sender’s identity $ID_{Snd}$, the algorithm outputs the encryption key $ek_{ID_{Snd}}$.
- RKGen$\left(msk,t,ID_{Rev}\right)\to ek_{ID_{Rev}}$: After inputting the master secret key msk and the receiver identity $ID_{Rev}$, during time period *t*, the algorithm outputs the decryption key $dk_{ID_{Rev}}$, which is associated with the time.
- Enc$(ek_{ID_{Snd}},t,ID_{Rev},m)\to C$: Given mpk, the encryption key $ek_{ID_{Snd}}$, a period time *t*, a receiver identity $ID_{Rev}$, and the message *m*, the algorithm outputs the ciphertext *C*.
- Dec$(dk_{ID_{Rec}},ID_{Snd},C)\to m$: Given mpk, the decryption key $dk_{ID_{Rev}}$, a sender identity $ID_{Snd}$, and the ciphertext *C*, the algorithm outputs *m*.
- Trap$\left(dk_{ID_{Rev}}\right)\to td_{ID}$: It takes as input a portion of the receiver’s private key to compute the trapdoor corresponding to the ciphertext.
- Test$(C_{ID_{i}},td_{ID_{i}},C_{ID_{j}},td_{ID_{j}})\to result$: This algorithm takes a receiver’s ciphertext with trapdoor $td_{ID_{i}}$ and another receiver’s ciphertext with trapdoor $td_{ID_{j}}$ as inputs and outputs a result of 0 or 1.

Correctness of RIBME-ET scheme: RIBME-ET Π = (Setup, SKGen, RKGen, Enc, Dec, Trap, Test) is correct if ∀λ∈N, (mpk,msk)← Setup($1^{\lambda })$, *Pr*[Dec$(dk_{ID_{Rev}}$, snd, Enc$\left(ek_{ID_{Snd}},t,rcv,m))=m\right]$≥1−negl(λ), and Test$\left(C_{ID_{i}},td_{ID_{i}},C_{ID_{j}},td_{ID_{j}}\right)$ = 0 or 1, where $ek_{ID_{Snd}},dk_{ID_{Rev}},$ and $td_{ID}$ are generated by SKGen($msk,ID_{Snd}$), RKGen($msk,t,ID_{Rev}$), and Trap$\left(dk_{ID_{Rev}}\right)$.

### 4.3. Security Definitions of RIBME-ET

We analyze the security model of the RIBME-ET scheme and define three types of adversaries under our system model:

- Type-I adversary: A Type-I adversary $A_{1}$, who possesses a trapdoor, attempts to recover the plaintext *m* from a ciphertext. Security against $A_{1}$ is defined as one-wayness under chosen identity and chosen ciphertext attacks (OW-ID-CCA).
- Type-II adversary: A Type-II adversary $A_{2}$, who has no trapdoor, attempts to determine which plaintext corresponds to a given ciphertext. Security against $A_{2}$ requires indistinguishability under chosen identity and chosen ciphertext attacks (IND-ID-CCA).
- Type-III adversary: A Type-III adversary $A_{3}$ tries to forge a ciphertext *C* corresponding to the sender’s identity. The forgery (C,Rev,Snd) is considered valid if for all encryption keys $ek_{ID_{Snd}}$ obtained by the adversary $A_{3}$ it holds that $ID_{Snd}=Snd$ and the identity Rev is not held by the adversary $A_{3}$. Security against Type-III adversary is existential unforgeability against identity under chosen message attacks (EU-ID-CMA).

**Definition** **1** (OW-ID-CCA)**.** *Regarding $A_{1}$, the RIBME-ET scheme meets OW-ID-CCA security when no PPT $A_{1}$ is winning the game below with a non-negligible advantage.*

1. Setup: Challenger C takes the security parameter λ as input, runs RIBME-ET.Setup($1^{\lambda })\to$mpk, and sends the master public key mpk to $A_{1}$.
2. Phase 1: $A_{1}$ may query the following oracles polynomially many times adaptively and in any order: $O_{SKGen},O_{RKGen},O_{Dec}$, and $O_{Trap}$.
  - $O_{SKGen}$: Given the identity of the sender Snd is received, $A_{1}^{\prime }$ answers the encryption key $ek_{Snd}$.
  - $O_{RKGen}$: Given the identity of the receiver Rev is received, $A_{1}^{\prime }$ answers the decryption key $dk_{Rev}$.
  - $O_{Dec}$: Given the identity of the receiver Rev, the identity of the target sender snd, and the ciphertext *C* are received, C answers the result of $Dec(mpk,dk_{Rev},Snd,C)$.
  - $O_{Trap}$: Given the identity of the target sender Snd and the identity of the receiver Rev are received, C answers the corresponding trapdoor td(Snd,Rev).
3. Challenge: $A_{1}$ sends identities $Snd^{*}$ and $Rev^{*}$ to C. Subsequently, C randomly chooses a message $m^{*}\in {\left\{0,1\right\}}^{n}$ in answer to $A_{1}$ with the challenge ciphertext *C* = $Enc(mpk,ek_{Snd^{*}},Rev^{*},t^{*},m^{*})$.
4. Phase 2: $A_{1}$ makes queries like in Phase 1.
5. Guess: $A_{1}$ answers a guess $m^{\prime }$.

We say that the adversary $A_{1}$ wins if $m^{\prime }=m^{*}$ in the above game, and the advantage of $A_{1}$ is defined as $adv_{RIBME-ET,A_{1}}^{OW-ID-CCA}\left(\lambda \right)=Pr\left(m^{\prime }=m^{*}\right)$.

In the above game, $A_{1}$ cannot ask the following queries:

$O_{RKGen}\left(Rev^{*}\right)$ and $O_{Dec}\left(Rev^{*},Snd^{*},C^{*}\right)$.

**Definition** **2** (IND-ID-CCA)**.** *We say that a RIBME-ET scheme is IND-ID-CCA-secure against Type-II adversaries if for any PPT adversary $A_{2}$, the advantage of $A_{2}$ in the following game with the challenger C is negligible in terms of the security parameter λ.*

1. Setup: This step is the same as that of the OW-ID-CCA security game in Definition 1.
2. Phase 1: This step is the same as that of the OW-ID-CCA security game in Definition 1 except for $O_{Trap}$.
3. Challenge: $A_{2}$ sends $snd^{*}$ and $rcv^{*}$ and selected messages $m_{0}^{*},m_{1}^{*}\in {\left\{0,1\right\}}^{n}$ of the same length to C. C selects a random bit b∈{0,1}, runs $RIBME-ET.Enc\left(ek_{ID_{Snd}},t,ID_{Rev},m_{b}\right)\to C_{b}$, and sends $C_{b}$ to $A_{2}$.
4. Phase 2: C responds to $A_{2}$’s queries as in Phase 1.
5. Guess: $A_{2}$ outputs $b^{\prime }\in${0,1}.

We say that the adversary $A_{2}$ wins if $b^{\prime }=b$ in the above game, and the advantage of $A_{2}$ is defined as $adv_{RIBME-ET,A_{2}}^{IND-ID-CCA}\left(\lambda \right)=\left|Pr\left(b^{\prime }=b\right)-\frac{1}{2}\right|$.

In the above game, $A_{2}$ cannot ask the following queries: $O_{RKGen}\left(Rev^{*}\right),O_{Trap}(Snd^{*},$

$Rev^{*}),$ and $O_{Dec}\left(Rev^{*},Snd^{*},C^{*}\right)$.

**Definition** **3** (EU-ID-CMA)**.** *We say that a RIBME-ET scheme is EU-ID-CMA-secure against Type-III adversaries if for any PPT adversary $A_{3}$, the advantage of $A_{3}$ in the following game with the challenger C is negligible in terms of the security parameter λ.*

1. Setup: This step is the same as that of the OW-ID-CCA security game in Definition 1.
2. Phase 1: This step is the same as that of the OW-ID-CCA security game in Definition 1 except for $O_{Dec}$ and $O_{Trap}$.
3. Forgery: $A_{3}$ outputs a forged ciphertext $C=(C,ID_{Rev},ID_{Snd})$ to C, C runs the ReceiverKey generation to obtain $dk_{Rev}$, C uses $dk_{Rev}$ to decrypt ciphertext *C*, and C outputs *m*. If Snd∀$Q_{O_{SKGen}}$, $Snd\neq ID_{Snd}$∧$Rev\notin Q_{O_{RKGen}}$∧m≠⊥, the adversary $A_{3}$ wins and C returns 1. Else, if the adversary $A_{3}$ fails, C returns 0.

We say that the adversary $A_{3}$ wins if C outputs *m* and returns 1 in the above game; the advantage of $A_{3}$ is defined as $adv_{RIBME-ET,A_{3}}^{EU-ID-CMA}\left(\lambda \right)=Pr\left[A_{3}wins\right]$.

## 5. Construction of RIBME-ET

In this section, we provide a RIBME-ET construction, and the scheme coincides with our system model. The concrete scheme is as follows:

- Setup: When the security parameter λ is input, the algorithm outputs a time period *t*$\in {\left\{0,1\right\}}^{*}$ and a bilinear group $\left(p,e,G_{1},G_{2}\right)$ with a generator $g\in G_{1}$, where the order of $G_{1}$ and $G_{2}$ is *p*. Then it randomly selects seven cryptographic hash functions $H_{0}:{\left\{0,1\right\}}^{*}\times {\left\{0,1\right\}}^{*}\to G_{1}$, $H_{1}:{\left\{0,1\right\}}^{*}\to G_{1}$, $H_{2}:G_{2}\to {\left\{0,1\right\}}^{l}$, $H_{3}:{\left\{0,1\right\}}^{n+\gamma }\to Z_{p}^{*}$, $H_{4}:G_{2}\to G_{1}$, $H_{5}:{\left\{0,1\right\}}^{n}\to G_{1}$, and $H_{6}:G_{1}\to {\left\{0,1\right\}}^{l}$, modeled as random oracles, and a polynomial-time computable padding function φ:${\left\{0,1\right\}}^{n}$→${\left\{0,1\right\}}^{l}$. We require that for all *m*∈${\left\{0,1\right\}}^{n}$, one can verify in polynomial time if *m* has been padded correctly and that φ(m) is efficiently invertible. In addition, it also selects two random numbers *r, s*∈$Z_{p}^{*}$ and computes $g_{0}=g^{r}$. Finally, the master public key and master secret key are $mpk=(g,g_{0},p,e,G_{1},G_{2},H_{0},H_{1},H_{2},H_{3},H_{4},H_{5},H_{6},\varphi )$ and msk=(r,s).
- Sender Key generation: Given the master key pair (mpk,msk) and a sender’s identity $ID_{snd}$→${\left\{0,1\right\}}^{*}$, the algorithm outputs the sender’s encryption key $ek_{ID_{Snd}}$ = $H_{1}{\left(ID_{Snd}\right)}^{s}$.
- Receiver Key generation: Given the master key pair (mpk,msk) and a receiver’s identity $ID_{Rev}$→${\left\{0,1\right\}}^{*}$, with a period of time *t*, the algorithm outputs the receiver’s decryption key $dk_{ID_{Rev}}=(dk_{ID_{Rev}}^{1}$, $dk_{ID_{Rev}}^{2}$, $dk_{ID_{Rev}}^{3}$)=($H_{0}{\left(ID_{Rev},t\right)}^{r}$, $H_{0}{\left(ID_{Rev},t\right)}^{s}$, $H_{0}\left(ID_{Rev},t\right)$).
- Encryption: Given the master public key mpk, the encryption key $ek_{ID_{Snd}}$, a time period *t*, the receiver’s identity $ID_{Rev}$, and a message *m*∈${\left\{0,1\right\}}^{n}$, the algorithm proceeds as follows:
  1. Sample random *a, b* ∈$Z_{p}^{*}$, and $z\in {\left\{0,1\right\}}^{\gamma }$.
  2. Compute *$C_{0}=g^{b}$ and $C_{1}=g^{a}$.*
  3. Compute *$k_{1}$ = $e(H_{0}\left(ID_{Rev},t\right),g_{0}^{a})$ and $k_{2}$ = $e(H_{0}\left(ID_{Rev},t\right),C_{0}\cdot ek_{ID_{Snd}})$.*
  4. Compute $C_{2}=g^{H_{3}\left(m,z\right)}$ and $C_{3}$ = φ(m)⊕$H_{2}\left(k_{1}\right)$⊕$H_{2}\left(k_{2}\right)$⊕$H_{6}\left(C_{2}\right)$.
  5. Compute $C_{4}=H_{5}{\left(m\right)}^{H_{3}\left(m,z\right)}\cdot H_{4}\left(e\left(g,H_{1}\left(ID_{Snd}\right)\cdot H_{0}\left(ID_{Rev},t\right)\right)\right)$.
  6. Output ciphertext $C=(C_{0},C_{1},C_{2},C_{3},C_{4})$.
- Decryption: Given the master public key mpk, a decryption key $dk_{ID_{Rev}}$, a target identity $ID_{Snd}=Snd$, and ciphertext $C=(C_{0},C_{1},C_{2},C_{3},C_{4})$, the algorithm proceeds as follows:
  1. Parse $C=(C_{0},C_{1},C_{2},C_{3},C_{4})$.
  2. Compute $k_{1}$ = $e(dk_{ID_{Rev}}^{1},C_{1})$ and $k_{2}$ = $e\left(dk_{ID_{Rev}}^{2},H_{1}\left(ID_{Snd}\right)\right)\cdot e\left(dk_{ID_{Rev}}^{3},C_{0}\right)$.
  3. Compute φ(m) = $C_{3}⊕H_{2}\left(k_{1}\right)⊕H_{2}\left(k_{2}\right)⊕H_{6}\left(C_{2}\right)$.
  4. If the padding is valid, return *m*. Otherwise, return ⊥.
- Trapdoor: The algorithm is performed by a receiver who takes $H_{1}\left(ID_{Snd}\right)$ and $dk_{ID_{Rev}}$ as input to produce the trapdoor. $td_{Rev}$ = $H_{1}\left(ID_{Snd}\right)\cdot dk_{ID_{Rev}}^{3}$= $H_{1}\left(ID_{Snd}\right)\cdot H_{0}\left(ID_{Rev},t\right)$. The scheme uses the decryption key of the receiver in the trapdoor generation phase, and the decryption key is divided into three components. The trapdoor generation phase uses one of the components to generate the trapdoor, which is not associated with the msk. Even if $dk_{ID_{Rev}}^{3}$ is leaked, it is not possible to compute the decryption key as a whole, and thus the use of a part of the decryption key has no effect on the security of the scheme.
- Test: This algorithm is performed by the cloud server, which takes two ciphertext–trapdoor pairs $\left(C_{A},td_{A}\right)$ and $\left(C_{B},td_{B}\right)$.
  $C_{A}=\left(C_{0A},C_{1A},C_{2A},C_{3A},C_{4A}\right)$,
  $C_{B}=\left(C_{0B},C_{1B},C_{2B},C_{3B},C_{4B}\right)$.
  1. Compute $\eta_{A}$ and $\eta_{B}$ as follows:
    $\eta_{A}$ = $\frac{C_{4A}}{H_{4}\left(e\left(g,td_{A}\right)\right)}$,
    $\eta_{B}$ = $\frac{C_{4B}}{H_{4}\left(e\left(g,td_{B}\right)\right)}$.
  2. Compute $e(C_{2A},\eta_{B})$ and $e(C_{2B},\eta_{A})$ as follows:
  Check whether $e\left(C_{2A},\eta_{B}\right)=e\left(C_{2B},\eta_{A}\right)$ holds. If it holds, output 1; otherwise, output 0.

Correctness:

1. For decryption, if $ID_{Snd}=Snd$, $ID_{Rev}=Rev$, and Snd matches Rev, the receiver will be able to decrypt the ciphertext. For a ciphertext $C=(C_{0},C_{1},C_{2},C_{3},C_{4})$ under an encryption of a message *m*, let $k_{1}^{\prime }$ and $k_{2}^{\prime }$ be the keys computed by the decryption algorithm.
  Compute $k_{1}$ = $e(dk_{ID_{Rev}}^{1},C_{1})$ = $e(H_{0}{\left(ID_{Rev},t\right)}^{r},g^{a})$ = $e(H_{0}\left(ID_{Rev},t\right),g^{ra})$ = $e(H_{0}\left(ID_{Rev},t\right),{g_{0}}^{a})$ = $k_{1}^{\prime }$.
  Compute $k_{2}$ = $e\left(dk_{ID_{Rev}}^{2},H_{1}\left(ID_{Snd}\right)\right)\cdot e\left(dk_{ID_{Rev}}^{3},C_{0}\right)$ = $e\left(H_{0}{\left(ID_{Rev},t\right)}^{s},H_{1}\left(ID_{Snd}\right)\right)\cdot e\left(H_{0}\left(ID_{Rev},t\right),g^{b}\right)$ = $e(H_{0}\left(ID_{Rev},t\right),g^{b}\cdot H_{1}{\left(ID_{Snd}\right)}^{s})$ = $k_{2}^{\prime }$.
  φ(m) = $C_{3}⊕H_{2}\left(k_{1}\right)⊕H_{2}\left(k_{2}\right)⊕H_{6}\left(C_{2}\right)$. Then return *m*.
2. For the equality test, if $ID_{Snd}=Snd_{A}\wedge ID_{Rev}=Rev_{A}\wedge ID_{Snd}=Snd_{B}\wedge ID_{Rev}=Rev_{B}$, $Snd_{A}$ matches $Rev_{A}$, and $Snd_{B}$ matches $Rev_{B}$, only receivers A and B can compute their respective trapdoors. For the ciphertext $C_{A}$ encrypted by message $m_{A}$ and the ciphertext $C_{B}$ encrypted by message $m_{B}$, we check the following:
  $$\eta_{A}=\frac{C_{4A}}{H_{4}\left(e\left(g,td_{A}\right)\right)}=H_{5}{\left(m_{A}\right)}^{H_{3}\left(m_{A},z_{A}\right)},$$
  $$\eta_{B}=\frac{C_{4B}}{H_{4}\left(e\left(g,td_{B}\right)\right)}=H_{5}{\left(m_{B}\right)}^{H_{3}\left(m_{B},z_{B}\right)},$$
  $$\begin{array}{cc} e(C_{2A},\eta_{B}) & =e(g^{H_{3}\left(m_{A},z_{A}\right)},H_{5}{\left(m_{B}\right)}^{H_{3}\left(m_{B},z_{B}\right)})\\ \; & =e(g,{H_{5}\left(m_{B}\right))}^{H_{3}\left(m_{A},z_{A}\right)H_{3}\left(m_{B},z_{B}\right)}, \end{array}$$
  $$\begin{array}{cc} e(C_{2B},\eta_{A}) & =e(g^{H_{3}\left(m_{B},z_{B}\right)},H_{5}{\left(m_{A}\right)}^{H_{3}\left(m_{A},z_{A}\right)})\\ \; & =e(g,{H_{5}\left(m_{A}\right))}^{H_{3}\left(m_{B},z_{B}\right)H_{3}\left(m_{A},z_{A}\right)}. \end{array}$$
  If $m_{A}=m_{B}$, then $e\left(C_{2A},\eta_{B}\right)=e\left(C_{2B},\eta_{A}\right)$, and it outputs 1; otherwise, it outputs 0.

## 6. Security Analysis of RIBME-ET

In this section, we demonstrate that our RIBME-ET construction is OW-ID-CCA-secure against a PPT Type-I adversary, IND-ID-CCA-secure against a PPT Type-II adversary, and EU-ID-CMA-secure against a PPT Type-III adversary.

### 6.1. OW-ID-CCA Security Against Type-I Adversary

As for confidentiality, we first look into the OW-ID-CCA security of our construction against a Type-I adversary. We change the Boneh–Franklin CCA-secure IBE’s proof method; our method of proof is similar to that of the IBME scheme. We prove that RIBME-ET is OW-ID-CCA-secure under the CBDH assumption. First, we define BPub^+^, a variant of BasicPub that is more suitable for our needs. BPub^+^ is composed of the following algorithms:

- Setup($1^{\lambda }$): Generate a symmetric pairing e: $G_{1}$×$G_{1}$→$G_{2}$, with $G_{1}$ and $G_{2}$ of an order *p* that depends on λ. Choose a random generator *g* of $G_{1}$. Sample a random $s\in Z_{q}^{*}$ and set $g_{0}=g^{s}$. Choose three hash functions: $H_{0}:G_{2}\to {\left\{0,1\right\}}^{n}$, $H_{1}:{\left\{0,1\right\}}^{n}\times {\left\{0,1\right\}}^{n}\to Z_{p}^{*}$, and $H_{2}:{\left\{0,1\right\}}^{n}\to {\left\{0,1\right\}}^{n}$, with $mpk=(p,e,n,g,g_{0},G_{1},G_{2},H_{0},H_{1},H_{2})$ and msk=s.
- KGen(mpk,msk): Choose a random $G\in G_{1}$. The public key is pk=G. The private key is $sk=G^{s}$.
- Enc(mpk,pk,m): To encrypt a message *m* under the public key pk=G, choose a random $\mu \in {\left\{0,1\right\}}^{n}$, set $y=H_{1}\left(\mu ,m\right)$, and output $C=(C_{0},C_{1},C_{2})$= $\left(g^{y},\mu ⊕H_{0}\left(e{\left(G,g_{0}\right)}^{y}\right)\right),m⊕H_{2}\left(\mu \right))$.
- Dec(mpk,sk,C): Let $C=(C_{0},C_{1},C_{2})$ be a ciphertext for the public key pk. To decrypt *C* using the private key sk, compute the following:
  1. $C_{1}⊕H_{0}\left(e\left(sk,C_{0}\right)\right)=\mu$.
  2. $C_{2}⊕H_{2}\left(\mu \right)=m$.
  3. Set $y=H_{1}\left(\mu ,m\right)$. Test that $C_{0}=g^{y}$. If not, reject the ciphertext.
  4. Output *m* as the decryption of *C*.

We show that if BPub^+^ is OW-CCA-secure, then our scheme is OW-ID-CCA-secure.

**Theorem** **1.** *Let the hash functions $H_{i}\left(i=0,1,2,3,4,5,6\right)$ be random oracles. Our construction is OW-ID-CCA-secure against a PPT Type-I adversary under the basis of the BDH assumption. More precisely, if $A_{1}$ can break our proposal with the advantage ε, suppose that $A_{1}$ makes at most $q_{S}$ sender key extraction queries, at most $q_{R}$ receiver key queries, at most $q_{D}$ decryption key queries, and at most $q_{T}$ trapdoor queries. We can conceive of a PPT algorithm $A_{1}^{\prime }$ to address the CBDH assumption with the advantage $\varepsilon^{\prime }\geq 512\varepsilon /e^{4}{\left(q_{R}+q_{S}+q_{D}+q_{T}+4\right)}^{4}q_{H_{2}}.$*

**Proof** **of** **Theorem** **1.** Given a CBDH assumption instance $\left(g,g^{a},g^{b},g^{c}\right)$, where $a,b,c\in Z_{p}^{*}$, the task of $A_{1}^{\prime }$ is to calculate $D=e{\left(g,g\right)}^{abc}$ by interacting with $A_{1}$ as shown below:

1. Setup: $A_{1}^{\prime }$ first performs the setup algorithm to generate $mpk=(g,g_{0},p,e,G_{1},G_{2},H_{0},H_{1},H_{2},H_{3},H_{4},H_{5},H_{6},\varphi )$, randomly selects $x,y\in Z_{p}^{*}$, and gives the mpk to $A_{1}$. $A_{1}^{\prime }$ then implicitly sets msk=(x,y), $g_{0}=g^{x}$, and $A_{1}^{\prime }$ has no knowledge about *x* and *y*. $A_{1}^{\prime }$ preserves the $L_{H_{i}}\left(i=0,1,2,3,4,5,6\right)$ lists to simulate the oracle $H_{i}$-query.
2. Phase 1: $A_{1}^{\prime }$ answers $A_{1}$’s queries.
  - $H_{0}$-query: On receiving this query with identity $ID_{Rev}$, if the query $ID_{Rev}$ is in a tuple $\left(ID_{Rev},Q,\beta ,b\right)\in L_{H_{0}}$, then *Q* is returned. Otherwise, $A_{1}^{\prime }$ randomly picks $\beta \in Z_{p}^{*}$ and inserts the new tuple $\left(ID_{Rev},Q,\beta ,b\right)$ into $L_{H_{0}}$, with a random coin b∈{0,1}, so that Pr[b=0]=ξ. If b=0, $A_{1}^{\prime }$ sets $Q=g^{\beta }$; otherwise, $A_{1}^{\prime }$ sets $Q=g^{c\beta }$. Finally, $\left(ID_{Rev},Q,\beta ,b\right)$ is added to $L_{H_{0}}$, and $A_{1}^{\prime }$ sends *Q* to $A_{1}$.
  - $H_{1}$-query: On receiving this query with identity $ID_{Snd}$, if the query $ID_{Snd}$ is in a tuple $\left(ID_{Snd},Q,\beta ,b\right)\in L_{H_{1}}$, then *Q* is returned. Otherwise, $A_{1}^{\prime }$ randomly picks $\beta \in Z_{p}^{*}$ and inserts the new tuple $\left(ID_{Snd},Q,\beta ,b\right)$ into $L_{H_{1}}$, with a random coin b∈{0,1}, so that Pr[b=0]=ξ. If b=0, $A_{1}^{\prime }$ sets $Q=g^{\beta }$; otherwise, $A_{1}^{\prime }$ sets $Q=g^{a\beta }$. Finally, $\left(ID_{Snd},Q,\beta ,b\right)$ is added to $L_{H_{0}}$, and $A_{1}^{\prime }$ sends *Q* to $A_{1}$.
  - $H_{2}$-query: $A_{1}^{\prime }$ maintains a list $L_{H_{2}}$ that stores tuples of the form $\left(X,h_{2}\right)$ with the history of calls to $L_{H_{2}}$. If the query *X* has already been completed, the challenger returns the value $h_{2}$. If not, it samples a random $h_{2}\in {\left\{0,1\right\}}^{n}$, adds $\left(X,h_{2}\right)$ to the list $L_{H_{2}}$, and returns $h_{2}$.
  - $H_{3}$-query: $A_{1}^{\prime }$ maintains a list $L_{H_{3}}$ that stores tuples of the form $\left(m,u,h_{3}\right)$ with the history of calls to $L_{H_{3}}$. If the queries *m* and *u* have already been completed, the challenger returns the value $h_{3}$. If not, it randomly picks *m*∈${\left\{0,1\right\}}^{n}$, *u*∈${\left\{0,1\right\}}^{n}$, and $h_{3}\in {\left\{0,1\right\}}^{\gamma }$ and inserts the new tuple $\left(m,u,h_{3}\right)$ into $L_{H_{3}}$. Subsequently, $A_{1}^{\prime }$ sends $h_{3}$ to $A_{1}$.
  - $H_{4}$-query: $A_{1}^{\prime }$ maintains a list $L_{H_{4}}$ that stores tuples of the form $\left(g_{h_{4}},h_{4}\right)$ with the history of calls to $L_{H_{4}}$. If the query $g_{h_{4}}$ has already been completed, the challenger returns the value $g_{H_{4}}$. If not, it randomly picks $g_{h_{4}}\in G_{2}$ and $h_{4}\in G_{1}$ and inserts the new tuple $\left(g_{h_{4}},h_{4}\right)$ into $L_{H_{4}}$. Subsequently, $A_{1}^{\prime }$ sends $h_{4}$ to $A_{1}$.
  - $H_{5}$-query: $A_{1}^{\prime }$ maintains a list $L_{H_{5}}$ that stores tuples of the form $\left(m,h_{5}\right)$ with the history of calls to $L_{H_{5}}$. If the query *m* has already been completed, the challenger returns the value $h_{5}$. If not, it randomly picks *m*∈${\left\{0,1\right\}}^{n}$ and $h_{5}\in G_{1}$ and inserts the new tuple $\left(m,h_{5}\right)$ into $L_{H_{5}}$. Finally, $A_{1}^{\prime }$ sends $h_{5}$ to $A_{1}$.
  - $H_{6}$-query: $A_{1}^{\prime }$ maintains a list $L_{H_{6}}$ that stores tuples of the form $\left(g_{H6},h_{6}\right)$ with the history of calls to $L_{H_{6}}$. If the query $g_{H6}$ has already been completed, the challenger returns the value $h_{6}$. If not, it randomly picks $g_{H6}\in G_{1}$ and $h_{6}\in {\left\{0,1\right\}}^{l}$ and inserts the new tuple $\left(g_{H6},h_{6}\right)$ into $L_{H_{6}}$. Finally, $A_{1}^{\prime }$ sends $h_{6}$ to $A_{1}$.
  - $O_{SKGen}$: $A_{1}^{\prime }$ performs a simulation algorithm with the $H_{1}$-query. Let $ID_{Snd}$ be the input of $O_{SKGen}$. $A_{1}^{\prime }$ obtains $H_{1}\left(Snd\right)=Q$. There is a tuple $\left(ID_{Snd},Q,\beta ,b\right)$ in $L_{H_{1}}$. If b=1, $A_{1}^{\prime }$ aborts; otherwise, it returns $ek_{Snd}=g^{y\beta }$.
  - $O_{RKGen}$: $A_{1}^{\prime }$ performs a simulation algorithm with the $H_{0}$-query. Let $ID_{Rev}$ be the input of $O_{RKGen}$. $A_{1}^{\prime }$ obtains $H_{0}\left(Rev,t\right)=Q$. There is a tuple $\left(ID_{Rev},Q,\beta ,b\right)$ in $L_{H_{0}}$. If b=1, $A_{1}^{\prime }$ aborts; otherwise, it returns $dk_{Rev}=\left(g^{x\beta },g^{y\beta },Q=g^{\beta }\right)$.
  - $O_{Dec}$: Let $ID_{Snd}=Snd$. $A_{1}^{\prime }$ performs a simulation algorithm to query the $H_{0}$-query and $H_{1}$-query. $A_{1}^{\prime }$ obtains the ciphertexts $C=(C_{0},C_{1},C_{2},C_{3},C_{4})$. $A_{1}^{\prime }$ sends the output of $Dec(mpk,dk_{Rev},Snd,C)$ to $A_{1}$.
    When $rev\neq Rev^{*}$, $A_{1}^{\prime }$ can query $O_{RKGen}$ to obtain $dk_{Rev}$ and returns the outcome $Dec(mpk,dk_{Rev},snd,C)$.
    Otherwise, $A_{1}^{\prime }$ can query $O_{SKGen}$ to obtain $ek_{Snd}$ and compute $k_{2}$ = $e(Q^{\prime },C_{0}\cdot ek_{Snd})$. For each tuple $\left(X,h_{2}\right)$ in $L_{H_{2}}$ and $\left(g_{H6},h_{6}\right)$ in $L_{H_{6}}$, $A_{1}^{\prime }$ computes $\varphi (m^{\prime })$ = $C_{3}⊕h_{2}⊕h_{6}$ and $H_{3}\left(m^{\prime },u^{\prime }\right)$. If $C_{2}=g^{H_{3}\left(m^{\prime },u^{\prime }\right)}$ and there exists a tuple $\left(g_{h_{4}},h_{4}\right)$ in $L_{H_{4}}$ such that $C_{4}=H_{5}{\left(m^{\prime }\right)}^{H_{3}\left(m^{\prime },u^{\prime }\right)}\cdot h_{4}$ is valid, it returns $m^{\prime }$. When $L_{H_{4}}$ has no such tuple, it returns ⊥.
  - $O_{Trap}$: Let $snd=Snd_{i}$. $A_{1}^{\prime }$ performs a simulation algorithm to query $H_{0}$ and $H_{1}$. $A_{1}^{\prime }$ runs the receiver key queries on the ID to obtain $dk_{Rev}=(dk_{Rev}^{1}$, $dk_{Rev}^{2}$, $dk_{Rev}^{3}$)$=(g^{x\beta },g^{y\beta },Q=g^{\beta })$ and responds to $A_{1}$ with $td_{id}$. When $Snd=Snd^{*}$ or $Rev=Rev^{*}$, $A_{1}^{\prime }$ fails. Otherwise, $A_{1}^{\prime }$ sends $td_{id}$ to $A_{1}$.
3. Challenge: Algorithm $A_{1}$ outputs a pair of sender and receiver identities $\left(Snd^{*},Rev^{*}\right)$ to $A_{1}^{\prime }$. $A_{1}^{\prime }$ randomly select a message $m^{*}\in {\left\{0,1\right\}}^{n}$ and utilizes a simulation algorithm to query oracles $H_{0}$ and $H_{1}$. The challenge pair of sender and receiver identities do not appear in the receiver key queries of Phase 1. Now $A_{1}^{\prime }$ performs the following steps:
  (1) Compute $H_{0}\left(rev,t\right)=Q$ and $H_{1}\left(snd\right)=Q^{\prime }$. If both the tuples $\left(Rev,Q,\beta ,b\right)\in L_{H_{0}}$ and $\left(Snd,Q^{\prime },\beta^{\prime },b^{\prime }\right)\in L_{H_{1}}$ do not have coins *b* and $b^{\prime }$ equal to 1, $A_{1}^{\prime }$ aborts. If not, we know that $dk_{Rev}^{2}=g^{cy\beta }$ and $H_{1}\left(Snd\right)=g^{x\beta }$. Hence, $H_{2}\left(k_{2}\right)$ = $H_{2}\left(e\left(dk_{ID_{Rev}}^{2},H_{1}\left(ID_{Snd}\right)\right)\cdot e\left(dk_{ID_{Rev}}^{3},C_{0}\right)\right)$, where $e(dk_{ID_{Rev}}^{2},H_{1}\left(ID_{Snd}\right))$ = $e(g^{cy\beta },g^{x\beta^{\prime }})$ = $D^{\beta \beta^{\prime }}$, and $Q=dk_{Rev}^{3}$.
  (2) Parse $C=(C_{0},C_{1},C_{2},C_{3},C_{4})$, compute $L=1/(\beta \beta^{\prime })$, and take a random tuple $\left(X,h_{2}\right)$. Return $D^{\prime }={\left(X\cdot e{\left(Q,C_{0}\right)}^{-1}\right)}^{L}$.
4. Phase 2: $A_{1}$ makes queries like in Phase 1.
5. Guess: $A_{1}$ answers with a guess $m^{\prime }$ for $m^{*}$. $A_{1}^{\prime }$ answers with a CBDH solution $D^{\prime }$.

Analysis: First of all, note that the simulation is perfect since in the above game we require that the challenge (C,Rev,Snd) satisfies the condition that Rev disappears in $O_{RKGen}$, Rnd disappears in $O_{SKGen}$, and the challenge C disappears in $O_{Dec}$. Assuming that the adversary makes at most $q_{R},q_{S},q_{D},$ and $q_{T}$ queries for $O_{RKGen},O_{SKGen},O_{Dec},$ and $O_{Trap}$, the probability that $A_{1}^{\prime }$ does not abort for any of these calls is $\delta^{q_{R}+q_{S}+q_{D}+q_{T}}$. Similarly, the probability that $A_{1}^{\prime }$ does not abort overall is $\delta^{q_{R}+q_{S}+q_{D}+q_{T}}{\left(1-\delta \right)}^{4}$, which is maximized at $\delta_{opt}=\left(q_{R}+q_{S}+q_{D}+q_{T}\right)/\left(q_{R}+q_{S}+q_{D}+q_{T}+4\right)$. If we use $\delta_{opt}$ as the probability of obtaining coins b=0 in the $H_{0}$ and $H_{1}$ queries, we have that the probability of $A_{1}^{\prime }$ not aborting is at least $256/e^{4}{\left(q_{R}+q_{S}+q_{D}+q_{T}+4\right)}^{4}$.

If $A_{1}^{\prime }$ does not abort, it outputs the correct solution $D^{\prime }$ with a probability of at least $2\varepsilon /q_{H_{2}}$. Hence, $A_{1}^{\prime }$ solves the CBDH problem with an advantage of $512\varepsilon /e^{4}{\left(q_{R}+q_{S}+q_{D}+q_{T}+4\right)}^{4}q_{H_{2}}$. □

### 6.2. IND-ID-CCA Security Against Type-II Adversary

As for privacy, we look into the IND-ID-CCA security of our construction against a Type-II adversary.

**Theorem** **2.** *Let the hash functions $H_{i}\left(i=0,1,2,3,4,5,6\right)$ be random oracles. Our construction is IND-ID-CCA-secure against a PPT Type-II adversary under the basis of the CBDH assumption. More precisely, if $A_{2}$ can break our proposal with the advantage ε, suppose that $A_{2}$ makes at most $q_{S}$ sender key extraction queries, at most $q_{R}$ receiver key queries, and at most $q_{D}$ decryption key queries. We can conceive of a PPT algorithm $A_{2}^{\prime }$ to address the CBDH assumption with the advantage $\varepsilon^{\prime }\geq 54\varepsilon /e^{3}{\left(q_{R}+q_{S}+q_{D}+3\right)}^{3}q_{H_{2}}.$*

**Proof** **of** **Theorem** **2.** Given a CBDH assumption instance $\left(g,g^{a},g^{b},g^{c}\right)$, where $a,b,c\in Z_{p}^{*}$, the task of $A_{2}^{\prime }$ is to calculate $D=e{\left(g,g\right)}^{abc}$ by interacting with $A_{2}$ as shown below:

1. Setup: $A_{2}^{\prime }$ first performs the setup algorithm to generate $mpk=(g,g_{0},p,e,G_{1},G_{2},H_{0},H_{1},H_{2},H_{3},H_{4},H_{5},H_{6},\varphi )$, randomly selects $x,r\in Z_{p}^{*}$, and sends the mpk to $A_{2}$. $A_{2}^{\prime }$ implicitly sets msk=x and $g_{0}=g^{x}$, and $A_{2}^{\prime }$ has no knowledge about *x*. $A_{2}^{\prime }$ preserves the $L_{H_{i}}\left(i=0,1,2,3,4,5,6\right)$ lists to simulate the oracle $H_{i}$-query.
2. Phase 1: $A_{2}^{\prime }$ answers $A_{2}$’s queries.
  - $H_{0}$-query: On receiving this query with identity $ID_{Rev}$, if query $ID_{Rev}$ is in a tuple $\left(ID_{Rev},Q,\beta ,b\right)\in L_{H_{0}}$, then *Q* is returned. Otherwise, $A_{2}^{\prime }$ randomly picks $\beta \in Z_{p}^{*}$ and inserts the new tuple $\left(ID_{Rev},Q,\beta ,b\right)$ into $L_{H_{0}}$, with a random coin b∈{0,1}, so that Pr[b=0]=ξ. If b=0, $A_{2}^{\prime }$ sets $Q=g^{\beta }$; otherwise, $A_{2}^{\prime }$ sets $Q=g^{c\beta }$. Finally, $\left(ID_{Rev},Q,\beta ,b\right)$ is added to $L_{H_{0}}$, and $A_{2}^{\prime }$ sends *Q* to $A_{2}$.
  - $H_{1}$-query: $A_{2}^{\prime }$ maintains a list $L_{H_{2}}$ that stores tuples of the form $\left(Snd_{i},Y_{i}\right)$ with the history of calls to $L_{H_{2}}$. If the query $Snd_{i}$ has already been completed, the challenger returns the value $Y_{i}$. If not, it samples a random $Y_{i}\in G_{1}$, adds $\left(Snd_{i},Y_{i}\right)$ to the list, and returns $Y_{i}$.
  - $H_{2}$-query: $A_{2}^{\prime }$ maintains a list $L_{H_{2}}$ that stores tuples of the form $\left(X,h_{2}\right)$ with the history of calls to $L_{H_{2}}$. If the query *X* has already been completed, the challenger returns the value $h_{2}$. If not, it samples a random $h_{2}\in {\left\{0,1\right\}}^{n}$, adds $\left(X,h_{2}\right)$ to the list $L_{H_{2}}$, and returns $h_{2}$.
  - $H_{3}$-query: $A_{2}^{\prime }$ maintains a list $L_{H_{3}}$ that stores tuples of the form $\left(m,u,h_{3}\right)$ with the history of calls to $L_{H_{3}}$. If the queries *m* and *u* have already been, the challenger returns the value $h_{3}$. If not, it randomly picks *m*∈${\left\{0,1\right\}}^{n}$, *u*∈${\left\{0,1\right\}}^{n}$, and $h_{3}\in {\left\{0,1\right\}}^{\gamma }$ and inserts the new tuple $\left(m,u,h_{3}\right)$ into $L_{H_{3}}$. Subsequently, $A_{2}^{\prime }$ sends $h_{3}$ to $A_{2}$.
  - $H_{4}$-query: $A_{2}^{\prime }$ maintains a list $L_{H_{4}}$ that stores tuples of the form $\left(g_{h_{4}},h_{4}\right)$ with the history of calls to $L_{H_{4}}$. If the query $g_{h_{4}}$ has already been completed, the challenger returns the value $g_{H_{4}}$. If not, it randomly picks $g_{h_{4}}\in G_{2}$ and $h_{4}\in G_{1}$ and inserts the new tuple $\left(g_{h_{4}},h_{4}\right)$ into $L_{H_{4}}$. Subsequently, $A_{2}^{\prime }$ sends $h_{4}$ to $A_{2}$.
  - $H_{5}$-query: $A_{2}^{\prime }$ maintains a list $L_{H_{5}}$ that stores tuples of the form $\left(m,h_{5}\right)$ with the history of calls to $L_{H_{5}}$. If the query *m* has already been completed, the challenger returns the value $h_{5}$. If not, it randomly picks *m*∈${\left\{0,1\right\}}^{n}$ and $h_{5}\in G_{1}$ and inserts the new tuple $\left(m,h_{5}\right)$ into $L_{H_{5}}$. Finally, $A_{2}^{\prime }$ sends $h_{5}$ to $A_{2}$.
  - $H_{6}$-query: $A_{2}^{\prime }$ maintains a list $L_{H_{6}}$ that stores tuples of the form $\left(g_{H6},h_{6}\right)$ with the history of calls to $L_{H_{6}}$. If the query $g_{H6}$ has already been completed, the challenger returns the value $h_{6}$. If not, it randomly picks $g_{H6}\in G_{1}$ and $h_{6}\in {\left\{0,1\right\}}^{l}$ and inserts the new tuple $\left(g_{H6},h_{6}\right)$ into $L_{H_{6}}$, finally, $A_{2}^{\prime }$ sends $h_{6}$ to $A_{2}$.
  - $O_{SKGen}$: $A_{2}^{\prime }$ performs a simulation algorithm with the $H_{1}$-query. Let $ID_{Snd}$ be the input of $O_{SKGen}$. $A_{2}^{\prime }$ obtains $H_{1}\left(Snd\right)=Y$. There is a tuple $\left(Snd_{i},Y_{i}\right)$ in $L_{H_{1}}$, and it returns $Y_{i}^{r}$.
  - $O_{RKGen}$: $A_{2}^{\prime }$ performs a simulation algorithm with the $H_{0}$-query. Let $ID_{Rev}$ be the input of $O_{RKGen}$. $A_{2}^{\prime }$ obtains $H_{0}\left(Rev,t\right)=Q$. There is a tuple $\left(ID_{Rev},Q,\beta ,b\right)$ in $L_{H_{0}}$. If b = 1, $A_{2}^{\prime }$ aborts; otherwise, it returns $dk_{Rev}=\left(g_{0}^{\beta },Q^{r},Q=g^{\beta }\right)$.
  - $O_{Dec}$: Let $ID_{Snd}=Snd$. $A_{2}^{\prime }$ performs a simulation algorithm to query the $H_{0}$-query and $H_{1}$-query. $A_{2}^{\prime }$ obtains the ciphertexts $C=(C_{0},C_{1},C_{2},C_{3},C_{4})$. $A_{2}^{\prime }$ sends the output of $Dec(mpk,dk_{Rev},Snd,C)$ to $A_{2}$.
    When $Rev\neq Rev^{*}$, $A_{1}^{\prime }$ can query $O_{RKGen}$ to obtain $dk_{Rev}$ and returns the outcome $Dec(mpk,dk_{Rev},Snd,C)$.
    Otherwise, $A_{2}^{\prime }$ can query $O_{SKGen}$ to obtain $ek_{Snd}$ and compute $k_{2}$ = $e(Q^{\prime },C_{0}\cdot ek_{Snd})$. For each tuple $\left(X,h_{2}\right)$ in $L_{H_{2}}$ and $\left(g_{H6},h_{6}\right)$ in $L_{H_{6}}$, $A_{2}^{\prime }$ computes $\varphi (m^{\prime })$ = $C_{3}⊕h_{2}⊕h_{6}$ and $H_{3}\left(m^{\prime },u^{\prime }\right)$. If $C_{2}=g^{H_{3}\left(m^{\prime },u^{\prime }\right)}$ and there exists a tuple $\left(g_{h_{4}},h_{4}\right)$ in $L_{H_{4}}$ such that $C_{4}=H_{5}{\left(m^{\prime }\right)}^{H_{3}\left(m^{\prime },u^{\prime }\right)}\cdot h_{4}$ is valid, $m^{\prime }$ is returned. When $L_{H_{4}}$ has no such tuple, ⊥ is returned.
3. Challenge: Algorithm $A_{2}$ outputs equal-length messages $m_{0},m_{1}\in {\left\{0,1\right\}}^{n}$ along with the two pairs of sender and receiver identities $\left(Snd_{0},Rev_{0},Snd_{1},Rev_{0}\right)$ to $A_{2}^{\prime }$. The pairs of sender and receiver identities do not appear in the receiver key queries of Phase 1. Afterwards, $A_{2}^{\prime }$ utilizes a simulation algorithm to query the oracles $H_{0}$ and $H_{1}$. $A_{2}^{\prime }$ performs the following steps:
  (1) After selecting $Rev_{0}$ and $Rev_{1}$, $A_{2}^{\prime }$ queries $H_{0}\left(Rev_{0}\right)=Q_{0}$ and $H_{0}\left(Rev_{1}\right)=Q_{1}$. If the tuples $\left(ID_{Rev_{0}},Q_{0},\beta_{0},1\right)$ and $\left(ID_{Rev_{1}},Q_{1},\beta_{1},1\right)$ do not belong to $L_{H_{0}}$, $A_{2}^{\prime }$ aborts. Otherwise, we know that $b_{0}$ = 0 and $b_{1}$ = 1, which means that $Q_{0}=ek_{0}$ and $Q_{1}=ek_{1}$.
  (2) $A_{2}^{\prime }$ computes $C_{0}=g^{d}$ for a random $d\in Z_{p}^{*}$ and queries $H_{1}\left(Snd_{0}\right)=Y_{0}$ and $H_{1}\left(Snd_{1}\right)=Y_{1}$. It uses them to obtain $\varphi (m_{0}^{\prime })$ = $C_{3}⊕H_{2}\left(Q_{0},C_{0}\cdot Y_{1}^{r}\right)⊕H_{6}\left(g_{H6}\right)$ and $\varphi (m_{1}^{\prime })$ = $C_{3}⊕H_{2}\left(Q_{1},C_{0}\cdot Y_{1}^{r}\right)⊕H_{6}\left(g_{H6}\right)$. Note that $ek_{Snd}=Y_{i}^{r}$.
  (3) $A_{2}^{\prime }$ sends $m_{0}^{\prime },m_{1}^{\prime },Q_{0},$ and $Q_{1}$ to its challenger and receives $C=(C_{1},C_{2},C_{3},C_{4})$ as a response.
  (4) $A_{2}^{\prime }$ computes $C^{\prime }=\left(C_{0},C_{1},C_{2},C_{3},C_{4}\right)$ and sends it to $A_{2}$. This is a proper encryption of $m_{b}$ under the condition $ID_{Rev_{b}}=Rev_{b}$ and sender’s identity $Snd_{b}$.
4. Phase 2: $A_{2}^{\prime }$ makes queries like in Phase 1.
5. Guess: $A_{2}$ answers with a guess $b^{\prime }\in \left\{0,1\right\}$. $A_{2}$ responds to its challenger with the same guess.

**Analysis**: Note that the simulation is perfect since in the above game we require that the challenge $\left(m_{0},m_{1},Rev_{0},Snd_{0},Rev_{1},Snd_{1}\right)$ satisfies the condition that $Rev_{b}$ disappears in $O_{RKGen}$ and $Snd_{b}$ disappears in $O_{SKGen}$; meanwhile, the encrypted challenge ciphertext cannot access $O_{Dec}$. Assuming that the adversary makes at most $q_{R},q_{S},$ and $q_{D}$ queries for $O_{RKGen},O_{SKGen},$ and $O_{Dec}$, the probability that $A_{2}^{\prime }$ does not abort for any of these calls is $\delta^{q_{R}+q_{S}+q_{D}}$. Similarly, the probability that $A_{2}^{\prime }$ does not abort overall is $\delta^{q_{R}+q_{S}+q_{D}}{\left(1-\delta \right)}^{3}$, which is maximized at $\delta_{opt}=\left(q_{R}+q_{S}+q_{D}\right)/\left(q_{R}+q_{S}+q_{D}+3\right)$. If we use $\delta_{opt}$ as the probability of obtaining coins b=0 in the $H_{0}$ and $H_{1}$ queries, we have that the probability of $A_{2}^{\prime }$ not aborting is at least $27/e^{3}{\left(q_{R}+q_{S}+q_{D}+3\right)}^{3}$.

If $A_{2}^{\prime }$ does not abort, it outputs the correct solution *D* with a probability of at least $2\varepsilon /q_{H_{2}}$. Hence, $A_{2}^{\prime }$ solves the CBDH problem with an advantage of $54\varepsilon /e^{3}{\left(q_{R}+q_{S}+q_{D}+3\right)}^{3}q_{H_{2}}$. □

### 6.3. EU-ID-CMA Security Against Type-III Adversary

As for the authenticity, we look into the EU-ID-CMA security of our construction against a Type-III adversary.

**Theorem** **3.** *Let the hash functions $H_{i}\left(i=0,1,2,3,4,5,6\right)$ be random oracles. Our construction is EU-ID-CMA-secure against a PPT Type-III adversary on the basis of the CBDH assumption. More precisely, if $A_{3}$ can break our proposal with the advantage ε, suppose that $A_{3}$ makes at most $q_{S}$ sender key extraction queries and at most $q_{R}$ receiver key queries. We can conceive of a PPT algorithm $A_{3}^{\prime }$ to address the CBDH assumption with the advantage $\varepsilon^{\prime }\geq 8\varepsilon /e^{2}{\left(q_{R}+q_{S}+2\right)}^{2}q_{H_{2}}.$*

The proof of this theorem is very similar with that of Theorem 1, which demonstrates the OW-ID-CCA security of our RIBME-ET scheme. Similarly to the proof of Theorem 1, we can prove the EU-ID-CMA security of our scheme. We provide the full proof of this theorem in Appendix A.

## 7. Performance Evaluation

In this section, we will evaluate the performance of our proposed scheme through theoretical comparisons and experimental evaluations to show its effectiveness and practicality.

### 7.1. Functionality and Security Comparisons

Table 2 shows the functionality and security comparisons between our RIBME-ET scheme and similar schemes. It is obvious that the IBME [12] scheme ensures the authenticity of data and privacy of users but does not provide equality test functionality or achieve CCA security. Since IBME [12] only implements CPA security, with valid plaintext and ciphertext pairs at the sender and receiver, an attacker can use it to forge any message, thus threatening the authenticity of the ciphertext in cloud storage.

The IBEET [10,18] scheme ensures data confidentiality but provides neither bilateral access control nor user revocation. The IBME-ET [17] scheme does not support user revocation. In the later comparison of the experimental analysis, our scheme is found to be more efficient than theirs. By comparing different schemes, our proposed RIBME-ET scheme can be found to realize all the functionality and security required, which not only ensures the confidentiality of data, privacy of users, user revocation, and bilateral access control and achieves CCA security but also provides equality test functionality for ciphertexts generated under different identities in smart healthcare.

Table 3 shows the computational cost comparison of our scheme with other schemes in terms of encryption key generation, decryption key generation, encryption, decryption, trapdoor generation, and equality testing. In Table 3, Exp is the exponentiation, *p* is the pairing, *H* and $H^{\prime }$ are hash-to-point operations in $G_{1}$ and $G_{2}$, and the symbol − indicates that the scheme does not have this phase. Our scheme has a lower computational cost compared to the IBME-ET [17] scheme in the decryption key generation, decryption, trapdoor generation, and equality test phases. Table 4 gives a comparison of the communication overhead of our scheme with that of other schemes, showing the results for encryption key, decryption key, trapdoor, and ciphertext comparisons. In Table 4, $|G_{1}|$ and $|G_{2}|$ are the sizes of the elements in groups $G_{1}$ and $G_{2}$, respectively. $|Z_{p}|$ is the size of the elements in $Z_{p}$, and λ is the security level. Our scheme has a lower communication cost in the trapdoor and ciphertext generation phases compared to the IBME-ET [17] scheme.

### 7.2. Experimental Analysis

**Figure 2:**
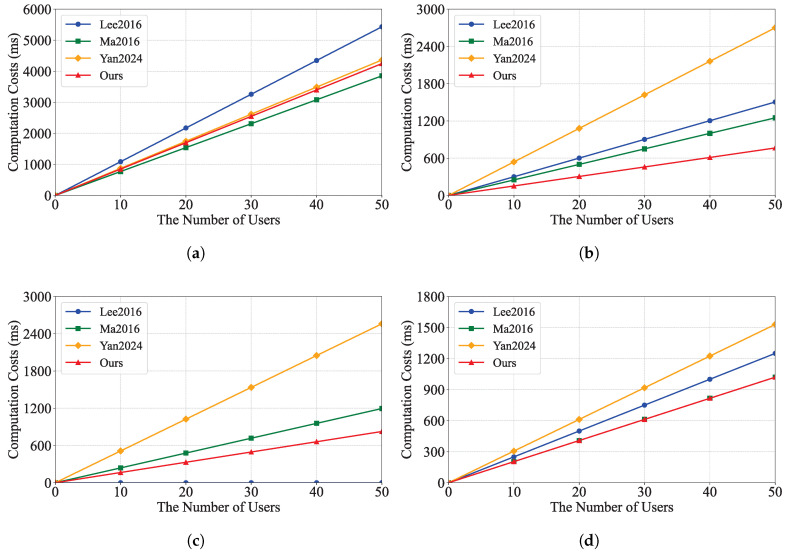
Computation cost of each scheme. (**a**) Encryption. (**b**) Decryption. (**c**) Trapdoor. (**d**) Test. These schemes: Lee2016 [10], Ma2016 [18], Yan2024 [17].

**Figure 3:**
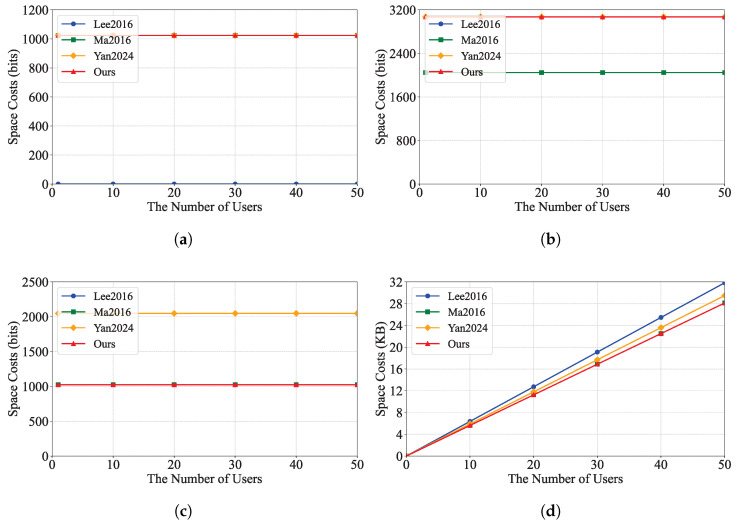
Communication cost of each scheme. (**a**) Encryption key. (**b**) Decryption key. (**c**) Trapdoor. (**d**) Ciphertext. These schemes: Lee2016 [10], Ma2016 [18], Yan2024 [17].

We evaluated the performance of our schemes by conducting extensive experiments. The tests were performed on a PC with the following specifications: Windows 10 Professional operating system, AMD Ryzen 5 5600 @ 3.5 GHz processor, and 32 GB RAM. The code was implemented in Java using the Java Pairing-Based Cryptography Library (JPBC) and tested on a 160-bit elliptic curve group constructed by the equation $y^{2}=x^{3}+x$ over a 512-bit field. We implemented the proposed scheme and compared it with IBEET [10,18] and IBME-ET [17]. The experimental results are shown in Figure 2 and Figure 3, which illustrate the computation costs and communication costs of these schemes, respectively.

Figure 2 shows the computational cost of each scheme. Our scheme has an advantage in the decryption and trapdoor generation phases, as it takes less time compared to the IBEET [10,18] and IBME-ET [17] schemes. In the equality test phase, our scheme takes the same amount of time as IBEET [18] and less time compared to IBEET [10] and IBME-ET [17]. Figure 3 shows the communication cost of each scheme and the communication cost used by our scheme in the trapdoor phase. Additionally, the ciphertext is the same as that of the IBME-ET scheme [18], which has a lower communication cost compared to the IBEET [10] and IBME-ET [17] schemes.

From Table 2, Table 3 and Table 4 and Figure 2 and Figure 3, we can conclude that, with a small sacrifice in computational and communication efficiency, our RIBME-ET scheme not only ensures the authenticity and confidentiality of data, the privacy of users, user revocation, and CCA security but also provides equality test functionality for ciphertexts generated under different identities in smart healthcare. Other related schemes cannot support this feature.

## 8. Conclusions and Future Work

In this paper, we introduce a new primitive RIBME-ET scheme, which not only ensures the privacy of users, the authenticity and confidentiality of data, and user revocation but also supports privacy-enhanced bilateral access control and provides equality test functionality for ciphertexts generated under different identities in smart healthcare. We proved the security of the scheme under random oracles. A comprehensive performance analysis shows the efficiency of our scheme. The limitation of our solution is that it is one-to-one for both the sender and the receiver. Considering specific smart healthcare scenarios, there may be a need for one-to-many data sharing and case matching between the sender and the receiver. Regarding future research, on the one hand, we can extend the scheme in the future to the case of fuzzy matching to further enhance the privacy of identities, as well as to mitigate key escrow and create an efficient infrastructure for key management and key revocation. On the other hand, we could consider increasing support for multi-user data sharing and case matching, extending the scheme to post-quantum security, and improving large-scale smart healthcare data integration.

## Figures and Tables

**Table 1:** Comparison of functionality.

Schemes	Equality Test	User Revocation	Bilateral Access Control	Security Model
PKEET [6]	✓ ^1^	×^ 2^	×	CCA
IBEET [10]	✓	×	×	CCA
IBME [12]	×	×	✓	CPA
IBME-ET [17]	✓	×	✓	CCA

^1^ ✓: Supports the functionality. ^2^ ×: Does not support the functionality.

**Table 2:** Comparison of functionality and security.

Schemes	Security	Equality Test	Authenticity	Privacy	User Revocation	Bilateral Access Control
IBEET [10]	CCA	✓ ^1^	×^2^	×	×	×
IBME [12]	CPA	×	✓	✓	×	✓
IBEET [18]	CCA	✓	×	×	×	×
IBME-ET [17]	CCA	✓	✓	✓	×	✓
Ours	CCA	✓	✓	✓	✓	✓

^1^ ✓: Supports the functionality. ^2^ ×: Does not support the functionality.

**Table 3:** Comparison of computation costs.

Schemes	SKGen	RKGen	Enc	Dec	Trap	Test
IBEET [10]	−	$3H^{\prime }+3Exp$	$3H^{\prime }+3p+6Exp$	3p+2Exp	0	2p+2Exp
IBEET [18]	*H*	H+2Exp	$2H^{\prime }+6Exp$	2p+2Exp	H+Exp	4p
IBME-ET [17]	H+Exp	$2H^{\prime }+3Exp$	$2H^{\prime }+2p+6Exp$	H+3p+3Exp	$H^{\prime }+p+4Exp$	6p
Ours	H+Exp	$H^{\prime }+3Exp$	$2H^{\prime }+3p+5Exp$	3p	*H*	4p

**Table 4:** Comparison of communication costs.

Schemes	Encryption Key	Decryption Key	Trapdoor	Ciphertext
IBEET [10]	−	$3|G_{2}|$	$|G_{2}|$	$4|G_{1}|+5\lambda$
IBEET [18]	$|G_{1}|$	$2|G_{1}|$	$|G_{1}|$	$4|G_{1}\left|+\right|Z_{p}|$
IBME-ET [17]	$|G_{1}|$	$3|G_{2}|$	$2|G_{2}|$	$3|G_{1}\left|+\right|G_{2}\left|+\right|Z_{p}|+\lambda$
Ours	$|G_{1}|$	$3|G_{2}|$	$|G_{2}|$	$4|G_{1}\left|+\right|Z_{p}|$

## Data Availability

The data used to support the findings of this study are included within the article.

## Appendix A. Proof of Theorem 3

The proof of Theorem 3 is very similar as that of Theorem 1. Let $A_{3}$ be a PPT Type-III adversary who breaks our proposal with the advantage ε. Suppose $A_{3}$ makes at most $q_{S}$ sender key extraction queries and at most $q_{R}$ receiver key queries. Then we can construct a PPT algorithm $A_{3}^{\prime }$ to address the CBDH assumption with the advantage $\varepsilon^{\prime }\geq 8\varepsilon /e^{2}{\left(q_{R}+q_{S}+2\right)}^{2}q_{H_{2}}.$

**Proof** **of** **Theorem** **3.** Given a CBDH assumption instance $\left(g,g^{a},g^{b},g^{c}\right)$, where $a,b,c\in Z_{p}^{*}$, the task of $A_{3}^{\prime }$ is to calculate $D=e{\left(g,g\right)}^{abc}$ by interacting with $A_{3}$ as shown below:

1. Setup: $A_{3}^{\prime }$ gives $A_{3}$ the public parameters $\left(g,g_{0}=g^{a},p,e,G_{1},G_{2},H_{0},H_{1},H_{2},H_{3},H_{4},H_{5},H_{6},\varphi \right)$, where $H_{i}\left(i=0,1,2,3,4,5,6\right)$ are random oracles controlled by $A_{3}^{\prime }$.
2. Phase 1: $A_{3}^{\prime }$ answers $A_{3}$’s queries.
  - $H_{0}$-query: On receiving this query with identity $ID_{Rev}$, if the query $ID_{Rev}$ is in a tuple $\left(ID_{Rev},Q,\beta ,b\right)\in L_{H_{0}}$, then it returns *Q*. Otherwise, $A_{3}^{\prime }$ randomly picks $\beta \in Z_{p}^{*}$ and inserts the new tuple $\left(ID_{Rev},Q,\beta ,b\right)$ into $L_{H_{0}}$, with a random coin b∈{0,1}, so that Pr[b=0]=ξ. If b = 0, $A_{3}^{\prime }$ sets $Q=g^{\beta }$; otherwise, $A_{3}^{\prime }$ sets $Q=g^{c\beta }$. Finally, $\left(ID_{Rev},Q,\beta ,b\right)$ is added to $L_{H_{0}}$, and $A_{3}^{\prime }$ sends *Q* to $A_{3}$.
  - $H_{1}$-query: On receiving this query with identity $ID_{Snd}$, if the query $ID_{Snd}$ is in a tuple $\left(ID_{Snd},Q,\beta ,b\right)\in L_{H_{1}}$, then it returns *Q*. Otherwise, $A_{3}^{\prime }$ randomly picks $\beta \in Z_{p}^{*}$ and inserts the new tuple $\left(ID_{Snd},Q,\beta ,b\right)$ into $L_{H_{1}}$, with a random coin b∈{0,1}, so that Pr[b=0]=ξ. If b = 0, $A_{3}^{\prime }$ sets $Q=g^{\beta }$; otherwise, $A_{3}^{\prime }$ sets $Q=g^{a\beta }$. Finally, $\left(ID_{Snd},Q,\beta ,b\right)$ is added to $L_{H_{0}}$, and $A_{3}^{\prime }$ sends *Q* to $A_{3}$.
  - $H_{2}$-query: $A_{3}^{\prime }$ maintains a list $L_{H_{2}}$ that stores tuples of the form $\left(X,h_{2}\right)$ with the history of calls to $L_{H_{2}}$. If the query *X* has already been completed, the challenger returns the value $h_{2}$. If not, it samples a random $h_{2}\in {\left\{0,1\right\}}^{n}$, adds $\left(X,h_{2}\right)$ to the list $L_{H_{2}}$, and returns $h_{2}$.
  - $H_{3}$-query: $A_{3}^{\prime }$ maintains a list $L_{H_{3}}$ that stores tuples of the form $\left(m,u,h_{3}\right)$ with the history of calls to $L_{H_{3}}$. If the queries *m* and *u* have already been completed, the challenger returns the value $h_{3}$. If not, it randomly picks *m*∈${\left\{0,1\right\}}^{n}$, *u*∈${\left\{0,1\right\}}^{n}$, and $h_{3}\in {\left\{0,1\right\}}^{\gamma }$ and inserts the new tuple $\left(m,u,h_{3}\right)$ into $L_{H_{3}}$. Subsequently, $A_{3}^{\prime }$ sends $h_{3}$ to $A_{3}$.
  - $H_{4}$-query: $A_{3}^{\prime }$ maintains a list $L_{H_{4}}$ that stores tuples of the form $\left(g_{h_{4}},h_{4}\right)$ with the history of calls to $L_{H_{4}}$. If the query $g_{h_{4}}$ has already been completed, the challenger returns the value $g_{H_{4}}$. If not, it randomly picks $g_{h_{4}}\in G_{2}$ and $h_{4}\in G_{1}$ and inserts the new tuple $\left(g_{h_{4}},h_{4}\right)$ into $L_{H_{4}}$. Subsequently, $A_{3}^{\prime }$ sends $h_{4}$ to $A_{3}$.
  - $H_{5}$-query: $A_{3}^{\prime }$ maintains a list $L_{H_{5}}$ that stores tuples of the form $\left(m,h_{5}\right)$ with the history of calls to $L_{H_{5}}$. If the query *m* has already been completed, the challenger returns the value $h_{5}$. If not, it randomly picks *m*∈${\left\{0,1\right\}}^{n}$ and $h_{5}\in G_{1}$ and inserts the new tuple $\left(m,h_{5}\right)$ into $L_{H_{5}}$. Finally, $A_{3}^{\prime }$ sends $h_{5}$ to $A_{3}$.
  - $H_{6}$-query: $A_{3}^{\prime }$ maintains a list $L_{H_{6}}$ that stores tuples of the form $\left(g_{H6},h_{6}\right)$ with the history of calls to $L_{H_{6}}$. If the query $g_{H6}$ has already been completed, the challenger returns the value $h_{6}$. If not, it randomly picks $g_{H6}\in G_{1}$ and $h_{6}\in {\left\{0,1\right\}}^{l}$ and inserts the new tuple $\left(g_{H6},h_{6}\right)$ into $L_{H_{6}}$. Finally, $A_{3}^{\prime }$ sends $h_{6}$ to $A_{3}$.
  - $O_{SKGen}$: $A_{3}^{\prime }$ performs a simulation algorithm with the $H_{1}$-query. Let $ID_{Snd}$ be the input of $O_{SKGen}$. $A_{3}^{\prime }$ obtains $H_{1}\left(Snd\right)=Q$. There is a tuple $\left(ID_{Snd},Q,\beta ,b\right)$ in $L_{H_{1}}$. If b = 1, $A_{3}^{\prime }$ aborts; otherwise, it returns $ek_{Snd}=g^{y\beta }$.
  - $O_{RKGen}$: $A_{3}^{\prime }$ performs a simulation algorithm with the $H_{0}$-query. Let $ID_{Rev}$ be the input of $O_{RKGen}$. $A_{3}^{\prime }$ obtains $H_{0}\left(Rev,t\right)=Q$. There is a tuple $\left(ID_{Rev},Q,\beta ,b\right)$ in $L_{H_{0}}$. If b = 1, $A_{3}^{\prime }$ aborts; otherwise, it returns $dk_{Rev}=\left(g^{x\beta },g^{y\beta },Q=g^{\beta }\right)$.
3. Forgery: Now $A_{3}$ sends (C,Rev,Snd) to $A_{3}^{\prime }$. Let $ID_{Snd}=Snd$. $A_{3}^{\prime }$ performs the following steps:
  (1) Compute $H_{0}\left(rev,t\right)=Q$ and $H_{1}\left(snd\right)=Q^{\prime }$. If both the tuples $\left(Rev,Q,\beta ,b\right)\in L_{H_{0}}$ and $\left(Snd,Q^{\prime },\beta^{\prime },b^{\prime }\right)\in L_{H_{1}}$ do not have coins *b* and $b^{\prime }$ equal to 1, $A_{1}^{\prime }$ aborts. If not, we know that $dk_{Rev}^{2}=g^{cy\beta }$ and $H_{1}\left(Snd\right)=g^{x\beta }$. Hence, $H_{2}\left(k_{2}\right)$ = $H_{2}\left(e\left(dk_{ID_{Rev}}^{2},H_{1}\left(ID_{Snd}\right)\right)\cdot e\left(dk_{ID_{Rev}}^{3},C_{0}\right)\right)$, where $e(dk_{ID_{Rev}}^{2},H_{1}\left(ID_{Snd}\right))$ = $e(g^{cy\beta },g^{x\beta^{\prime }})$ = $D^{\beta \beta^{\prime }}$, and $Q=dk_{Rev}^{3}$.
  (2) Parse $C=(C_{0},C_{1},C_{2},C_{3},C_{4})$, compute $L=1/(\beta \beta^{\prime })$, and take a random tuple $\left(X,h_{2}\right)$. Return $D^{\prime }={\left(X\cdot e{\left(Q,C_{0}\right)}^{-1}\right)}^{L}$.

Analysis: Note that the simulation is perfect since in the above game we require that the challenge $\left(C,Rev,Snd=ID_{Snd}\right)$ satisfies the conditions that Rev disappears in $O_{RKGen}$, Snd disappears in $O_{SKGen}$, and the challenge C disappears in $O_{Dec}$. Assuming that the adversary makes at most $q_{R}$ and $q_{S}$ queries for $O_{RKGen}$ and $O_{SKGen}$, the probability that $A_{3}^{\prime }$ does not abort for any of these calls is $\delta^{q_{R}+q_{S}}$. Similarly, the probability that $A_{3}^{\prime }$ does not abort at all is $\delta^{q_{R}+q_{S}}{\left(1-\delta \right)}^{2}$, which is maximized at $\delta_{opt}=\left(q_{R}+q_{S}\right)/\left(q_{R}+q_{S}+2\right)$. If we use $\delta_{opt}$ as the probability of obtaining coins b=0 in the $H_{0}$ and $H_{1}$ queries, we have that the probability of $A_{3}^{\prime }$ not aborting is at least $4/e^{2}{\left(q_{R}+q_{S}+2\right)}^{2}$. If $A_{3}^{\prime }$ does not abort, it outputs the correct solution $D^{\prime }$ with a probability of at least $2\varepsilon /q_{H_{2}}$. Hence, $A_{3}^{\prime }$ solves the CBDH problem with an advantage of $8\varepsilon /e^{2}{\left(q_{R}+q_{S}+2\right)}^{2}q_{H_{2}}$. □
